# Dynamic, Nondestructive Imaging of a Bioengineered Vascular Graft Endothelium

**DOI:** 10.1371/journal.pone.0061275

**Published:** 2013-04-09

**Authors:** Bryce M. Whited, Matthias C. Hofmann, Peng Lu, Yong Xu, Christopher G. Rylander, Ge Wang, Etai Sapoznik, Tracy Criswell, Sang Jin Lee, Shay Soker, Marissa Nichole Rylander

**Affiliations:** 1 School of Biomedical Engineering and Sciences, Virginia Tech–Wake Forest University, Blacksburg, Virginia, United States of America; 2 Department of Electrical and Computer Engineering, Virginia Polytechnic Institute and State University, Blacksburg, Virginia, United States of America; 3 Department of Mechanical Engineering, Virginia Polytechnic Institute and State University, Blacksburg, Virginia, United States of America; 4 Wake Forest Institute for Regenerative Medicine, Wake Forest University School of Medicine, Winston-Salem, North Carolina, United States of America; Instituto de Engenharia Biomédica, University of Porto, Portugal

## Abstract

Bioengineering of vascular grafts holds great potential to address the shortcomings associated with autologous and conventional synthetic vascular grafts used for small diameter grafting procedures. Lumen endothelialization of bioengineered vascular grafts is essential to provide an antithrombogenic graft surface to ensure long-term patency after implantation. Conventional methods used to assess endothelialization *in vitro* typically involve periodic harvesting of the graft for histological sectioning and staining of the lumen. Endpoint testing methods such as these are effective but do not provide real-time information of endothelial cells in their intact microenvironment, rather only a single time point measurement of endothelium development. Therefore, nondestructive methods are needed to provide dynamic information of graft endothelialization and endothelium maturation *in vitro*. To address this need, we have developed a nondestructive fiber optic based (FOB) imaging method that is capable of dynamic assessment of graft endothelialization without disturbing the graft housed in a bioreactor. In this study we demonstrate the capability of the FOB imaging method to quantify electrospun vascular graft endothelialization, EC detachment, and apoptosis in a nondestructive manner. The electrospun scaffold fiber diameter of the graft lumen was systematically varied and the FOB imaging system was used to noninvasively quantify the affect of topography on graft endothelialization over a 7-day period. Additionally, results demonstrated that the FOB imaging method had a greater imaging penetration depth than that of two-photon microscopy. This imaging method is a powerful tool to optimize vascular grafts and bioreactor conditions *in vitro*, and can be further adapted to monitor endothelium maturation and response to fluid flow bioreactor preconditioning.

## Introduction

Cardiovascular disease is currently the leading cause of death in the U.S., accounting for nearly 600,000 deaths in 2008 [1]. Atherosclerosis is one of the most prevalent and severe forms of cardiovascular disease and commonly treated using bypass surgery. The most widely used and reliable conduits for bypass surgery are autologous vessels, such as the saphenous vein and internal mammary artery [2]. Autologous vessels, however, still have a 35% failure rate and 5–30% of patients have no suitable vein or artery available due to vascular disease or other complications [3]. Fully synthetic grafts made from Dacron and expanded polytetrafluoroethylene have been successfully used for over 50 years for large diameter (>5 mm) bypass conduits, however, these grafts are susceptible to intimal hyperplasia and surface thrombogenicity when used for small diameter bypass (<5 mm) [4]. Therefore, small diameter vascular graft alternatives are greatly needed to address the shortcomings associated with autologous and fully synthetic grafts.

Bioengineering of small diameter vascular grafts holds great potential to address the shortage of bypass grafts. Bioengineered vascular grafts must be able to withstand physiological forces associated with blood flow, possess good suture retention, and maintain structural integrity during neotissue formation [5]. Additionally, the bioengineered graft must possess a lumen surface that inhibits thrombus formation, unwanted inflammatory responses, and limits proliferation of smooth muscle cells that may cause neointimal hyperplasia. In native vessels, endothelial cells (ECs) lining the lumen of blood vessels provide a nonthrombogenic barrier between circulating blood and the blood vessel wall, and play a key role in regulating dynamic mechanisms that protect blood vessels from the aforementioned detriments [6]. Previous studies have shown that incorporation of an EC monolayer on the graft lumen prevents thrombogenic events and contributes to long-term patency and function [7]–[10]. It is therefore broadly accepted that bioengineered vascular grafts must possess a confluent, healthy, and mature endothelium on the lumen surface of the graft to maintain graft patency and function [11].

A promising approach to obtain a bioengineered vascular graft with a functional endothelium is to seed the lumen with autologous ECs *in vitro*, harvested from a variety of sources such as veins [8], arteries [12], and circulating endothelial progenitor cells [13], prior to implantation. Successful graft endothelialization *in vitro* relies heavily on the ECs being able to attach, proliferate, and form a confluent endothelium on the graft lumen surface. Factors that affect graft endothelialization include the inherent material properties of the graft including biomaterial surface topography, chemistry, elasticity, and the ability to adsorb proteins [14]. The ability of ECs to strongly adhere to the surface of the graft surface is important because the hydrodynamic shear stress that they will experience by blood flow *in vivo* increases the chance of detachment and subsequent thrombus formation and vessel occlusion [11]. Several groups have shown that preconditioning the graft with a gradual increase in shear stress via fluid flow *in vitro* enhances overall EC retention under physiological flow [15]–[17]. Flow preconditioning of grafts in a well-controlled environment, such as fluid flow bioreactors, allows ECs to gradually adapt to the shear stress through reorganization of their cytoskeleton, presence of focal adhesions, and cell alignment with the direction of fluid flow to increase EC adhesion strength [18]–[20].

Experimental approaches to promote a confluent, adherent, and shear resistant endothelium in bioengineered vascular grafts, such as flow preconditioning, must be rigorously tested *in vitro* prior to implantation *in vivo*. Critical parameters that need to be measured to assess graft endothelialization include EC attachment, proliferation, and coverage of the lumen surface. Additionally, ensuring that ECs remain adherent to the lumen surface (resisting detachment) and determining their response to physiological fluid flow is of great importance to creating successful *in vitro* flow preconditioning methods and protocols. Current approaches used to assess these parameters include techniques such as histological sectioning and staining, which, aside from being time and labor intensive, destroys the graft and only provides a single time point measurement. However, endothelialization and EC response to flow mediated shear stress are dynamic processes and would be best understood if continuous observation of the lumen within an intact vessel were possible. Therefore, the ability to noninvasively image the growth, health, and integrity of a vascular graft endothelium during preconditioning in real-time is greatly needed and will aid in identification and optimization of scaffold properties and preconditioning protocols to enhance EC function and ultimately graft success once implanted.

The necessity to monitor vascular graft endothelialization and maturation in real time during preconditioning has led our group to develop a fiber optic based (FOB) imaging system to accomplish this task [21], [22]. The imaging system is designed to noninvasively assess the graft endothelium without disturbing the graft during preconditioning in a bioreactor. In this study, we evaluate the feasibility of the FOB imaging system to image and quantify endothelialization and EC detachment from an electrospun vascular graft in a dynamic and noninvasive manner. The electrospun fiber diameter of the graft lumen was systematically varied and the FOB imaging system was used to noninvasively quantify the affect of topography on graft endothelialization over a 7-day period. Additionally, the health of the endothelium was assessed by quantifying EC apoptosis on the lumen in response to varying levels of physiological insult. Finally, the imaging depth of the FOB imaging system was directly compared to that of two-photon fluorescence microscopy. The results of this study demonstrate the potential of the FOB imaging system to be utilized to nondestructively assess the maturation of a bioengineered vascular graft endothelium in real-time.

## Materials and Methods

### Vascular scaffold fabrication and micro-imaging channel (MIC) integration

**Figure 1:**
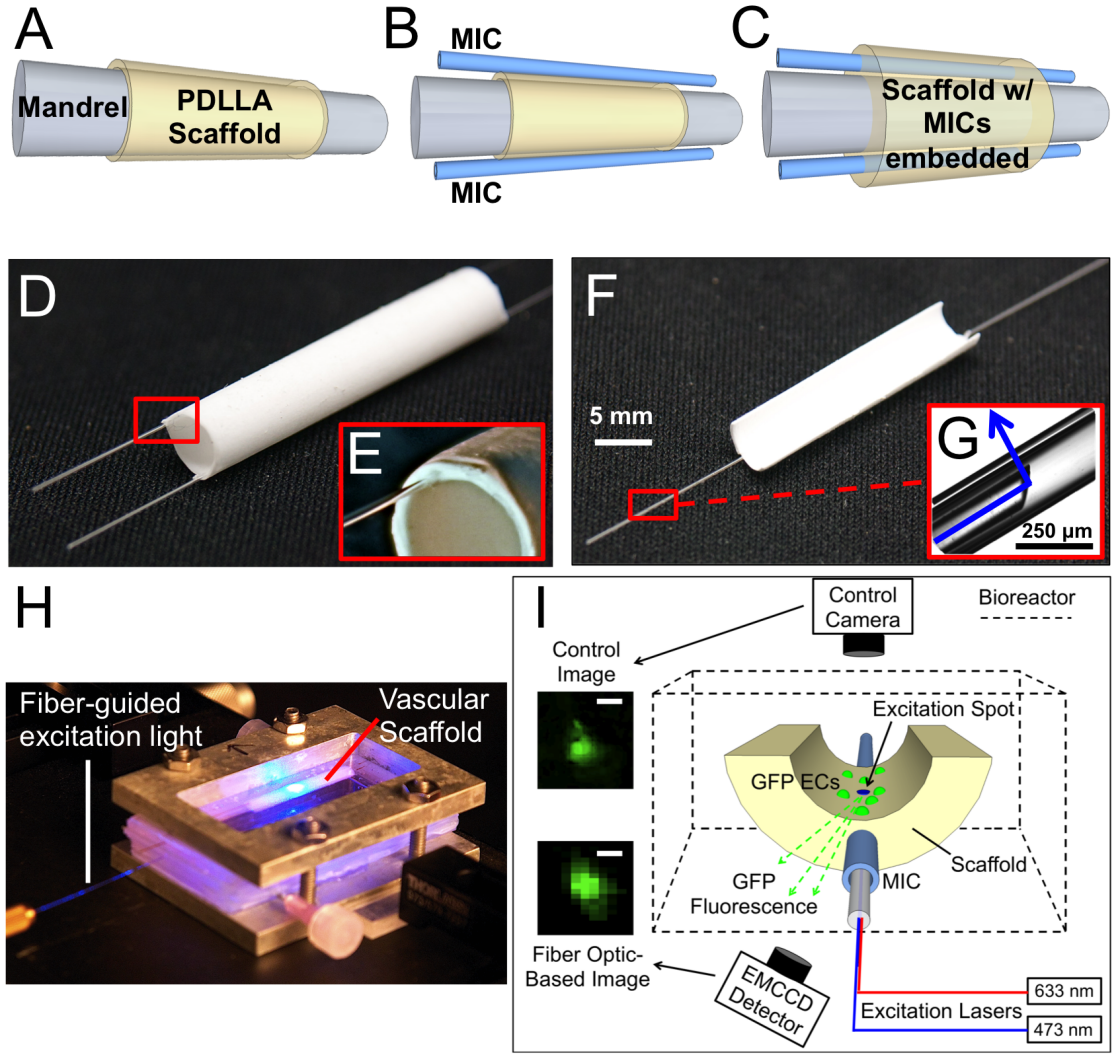
Vascular scaffold fabrication, MIC integration, and FOB imaging method. A–C) Illustration of the layer-by-layer electrospinning fabrication technique used to embed MICs within the wall of the vascular scaffold to facilitate imaging. D,E) Images of a vascular scaffold with MICs embedded within the wall. F) Image of a vascular scaffold sectioned lengthwise to validate endothelium imaging (half-vessel scaffold). G) Image of the fiber optic micro-mirror inserted within an MIC. Light is directed at a 90° angle relative to the fiber optic to allow excitation of ECs on the lumen of the vascular graft. H) Photograph of the vascular scaffold during an imaging experiment with the fiber-guided excitation light on. I) Schematic of the fiber optic imaging method used to create FOB images through the scaffold wall. Excitation laser light was guided to the fiber optic micro-mirror to excite GFP-ECs on the lumen of the vascular scaffold. The excitation spot created by the laser light was incrementally rastered on the lumen surface and GFP fluorescence was measured through the scaffold wall using an EMCCD detector. Linking the excitation spot to the GFP fluorescence values enabled fluorescence mapping to create a fiber optic-based (FOB) image. A corresponding control image was obtained by a direct-line-of-sight control camera to validate the imaging method on half-vessel scaffolds.

Vascular scaffolds with lumen diameter of 5 mm were fabricated by using a layer-by-layer electrospinning approach to integrate micro-imaging channels (MICs) directly into the wall of the scaffold. The MICs are silica glass capillaries with inner diameter of 150 µm and outer diameter of 245 µm (Polymicro Technologies, Phoenix, AZ) that facilitate insertion of fiber optics into the scaffold wall. To fabricate the scaffolds, solutions of poly (D,L-lactide) (PDLLA) (SurModics Pharmaceuticals, Birmingham, AL) with 5%, 10%, and 20% w/v concentrations were prepared in 1,1,1,3,3,3-hexafluoro-2-propanol (HFP, Sigma Aldrich, St. Louis, MO). Next, 150 µL of PDLLA was electrospun onto a rotating grounded mandrel (4.75 mm diameter) to form the scaffold lumen layer, as illustrated in Figure 1A. The 3 different concentrations of PDLLA were electrospun separately for the lumen layers, with varied electrospinning parameters (Table 1), to produce 3 different scaffolds with varying lumen fiber diameter and surface topography. Two MICs were then placed on opposite sides of the mandrel, as illustrated in Figure 1B. Next, 850 µL 20% w/v PDLLA was electrospun over the MICs to produce a composite vascular scaffold with MICs tightly embedded within the scaffold wall (Figure 1C). Figure 1D shows an entire vascular graft with 2 MICs firmly embedded into the vessel, and Figure 1E shows the MIC integrated within and extending from the vessel wall. A total of 13 vascular scaffolds exhibiting an average wall thickness of 510±55 µm were fabricated for this study. Vascular scaffold thicknesses were measured using a digital micrometer (Mitutoyo Corporation, Japan) before scaffold seeding.

**Table 1:** Electrospinning parameters used to fabricate vascular scaffolds with varying luminal fiber diameters.

	Polymer Concentration (% w/v)	Flow Rate (ml/hr)	Throw Distance (cm)	Voltage (kV)	Needle Gauge (g)	Electrospun Fiber Diameter ( µm)
Condition 1	5	1	7	20	30	0.36±0.07
Condition 2	10	2	10	15	25	1.00±0.31
Condition 3	20	5	15	12	18	3.32±0.63

Both full-vessel scaffolds (Figure 1D) and half-vessel scaffolds (Figure 1F) were fabricated and used for imaging in this experiment. Some vascular grafts were sectioned in half lengthwise (i.e. half-vessel scaffolds) before cell seeding to provide direct direct-line-of-sight viewing to the lumen portion of the graft for “control image” acquisition (Figure 1F). By taking direct-line-of-sight control images of the lumen with a conventional fluorescence microscope setup, we were able to validate the accuracy of the FOB imaging system by comparing the FOB images to their corresponding control image. It should be emphasized that these control images would otherwise be impossible to obtain unless the vessel were cut in half to allow direct-line-of-sight viewing of the lumen with the microscope. The half-vessel scaffolds were used to verify the accuracy of the FOB imaging system in quantifying EC coverage, cell detachment, and apoptosis (Section 2.5, 2.6 and 2.7; respectively). To acquire a control image, the lumen of a half-vessel scaffold was imaged with a CCD camera (XCD-X710, Sony, Japan) coupled with a 10× objective to obtain a corresponding direct-line-of-sight fluorescent image of the cells on the scaffold surface. After FOB imaging validation, we progressed to imaging full-vessel grafts to test the feasibility of nondestructively imaging and quantifying the effect of varied lumen topography on vascular graft endothelialization over time (Section 2.8 below).

### Scaffold Morphology

Scanning electron microscopy (SEM) was used to characterize electrospun scaffold morphology using a LEO 1550 field emission SEM (Carl Zeiss, Thornwood, NY). SEM images were analyzed with Image J software (U.S. National Institutes of Health, Bethesda, MD) to quantify scaffold fiber diameter. A total of 60 random fibers were selected from each image and measured to determine mean fiber diameter for each scaffold (n = 3 images/scaffold, n = 180 fibers total).

### Cell culture and scaffold seeding

A human dermal microvascular endothelial cell line (American Type Culture Collection, Manassas, VA) was infected with a lentivirus expressing green fluorescent protein (GFP) to stably express GFP, as previously described [21]. GFP-ECs were maintained in an incubator at 37°C, 5% CO_2_ and cultured in EGM-2 media (Lonza Biomedical, Walkersville, MD). Before cell seeding, the scaffolds were immersed in 70% ethanol for 5 min for sterilization and then washed 3 times for 5 min in sterile PBS to remove residual ethanol. GFP-ECs between passages 32–35 were then seeded onto the scaffolds at a density of 1.5×10^4^ cells/cm^2^, allowed to attach for 1 hr in the incubator, and rinsed once with PBS to remove unattached cells. Before imaging, the sample was placed in a custom bioreactor and filled with EGM-2 prior to imaging experiments. The bioreactor formed a hermetically sealed chamber for scaffold incubation while allowing fiber optic access through the MICs.

### Excitation light delivery and FOB image acquisition

Nondestructive imaging of the vascular lumen was carried out using the FOB imaging system, which enabled continuous image acquisition through the scaffold wall during construct maturation in the bioreactor. A succinct description of the FOB imaging method will follow, however, a more detailed description of the system and scanning/fluorescence mapping method can be found in our previously published papers [21], [22]. After the scaffold was seeded and the bioreactor assembled, the construct was placed on a heated imaging stage to maintain a 37°C environment (Figure 1H). Fluorescence excitation light was delivered to the scaffold lumen by inserting a 45° angle polished single-mode optical fiber (SMF430, Nufern Inc., East Granby, CT) into the MIC (Figure 1G). The angled fiber formed an optical micro-mirror by which excitation light could be launched at a 90° angle relative to the fiber longitudinal axis to illuminate GFP-ECs on the lumen of the vascular scaffold. A continuous wave solid-state laser with a wavelength of 473 nm (BLM-100, Extreme Lasers Inc., Seabrook, TX) was operated at 0.5 mW and coupled into the optical fiber with the micro-mirror tip to excite the cells on the vessel lumen.

To obtain an image of GFP-ECs seeded on the lumen surface, a fiber scanning and fluorescence mapping procedure was performed, as illustrated in Figure 1I. Briefly, the laser light introduced into the scaffold wall formed a localized beam of excitation light on the lumen surface, which will be referred to as the “excitation spot”. The excitation spot was then sequentially raster-scanned on the luminal surface by controlling the position and angle of the fiber micro-mirror within the MIC using two motion stages: a motorized translation stage (UTM100PP.1, Newport, Irvine, CA) and rotation stage (URM100APP, Newport). When the excitation spot reached a GFP-EC, the GFP was excited and subsequently emitted green fluorescence light, part of which traveled through the scaffold wall. The intensity of the fluorescence light leaving the scaffold was measured using an EM-CCD detector (iXon+, Andor, Belfast, Ireland) with a 2× long working distance objective attached (M-Plan, Mitutoyo). Bandpass filters (525/45 BrightLine, Semrock, Rochester, NY) were used to block excitation light while allowing GFP fluorescence to reach the EM-CCD detector. By coupling the fluorescence intensity to the excitation spot location, it was possible to create a fluorescence intensity map representing EC distribution on the lumen surface via signal processing using a custom MATLAB script (Mathworks, Cambridge, MA). This fluorescence intensity map will hereafter be referred to as the fiber optic based image, or FOB image. Custom LabView (National Instruments, Austin, TX) programs were written and utilized to control the motion stages and cameras for image acquisition.

### Nondestructive quantification of EC lumen coverage

The half-vessel scaffold was used to provide initial verification of the capability of the integrated FOB system to nondestructively image GFP-EC coverage of the vascular scaffold lumen during maturation in the bioreactor. Using the half-vessel configuration, we could simultaneously acquire a direct-line-of-sight “control” image of the lumen using conventional fluorescence microscopy (i.e. control camera) for comparison. After cell seeding, a 1 mm×250 µm area of the vascular lumen was scanned with the 473 nm laser excitation spot and GFP fluorescence intensities were recorded at each excitation spot location. The excitation spot was translated at 5 µm intervals and rotationally scanned at a step size of 1.33° to cover the aforementioned area. Three separate 1 mm×250 µm areas of the lumen were imaged daily using this method to follow the progression of endothelialization of the surface until confluency was achieved. For each FOB image obtained, a corresponding direct-line-of-sight control image was acquired with the control camera, to provide n = 3 FOB/control image pairs of the lumen at each time point, to assess the accuracy of the FOB imaging method. Both control and FOB images were then evaluated for area of EC coverage using Image J software. Images were imported into ImageJ, thresholded to the same value, a binary image was created, and then the area of cell coverage on the scaffold surface (fluorescence coverage) was measured.

### Nondestructive quantification of EC detachment

EC detachment from the lumen of vascular grafts was imaged and quantified after the scaffolds reached confluency. Half-vessel scaffolds were used for this study to expose the lumen to the control camera. At day 4 after seeding, the media in the bioreactor was replaced with a 1 µM trypsin EDTA solution in phosphate buffered saline (PBS) to induce EC detachment during imaging. A 250 µm×250 µm area of each scaffold lumen was continuously imaged after addition of trypsin for one hour using the FOB imaging system (n = 3 scaffolds total). Control images were also obtained for comparison to the FOB images. Control and FOB images were then analyzed, as explained previously, to nondestructively measure area of cell coverage through the scaffold wall over time.

### Nondestructive quantification of EC apoptosis

The FOB imaging method was used to nondestructively detect EC apoptosis on the vascular scaffold lumen. To induce varying degrees of EC apoptosis, concentrations of 1 and 5 µM camptothecin (MP Biomedicals, Solon, OH) were prepared in EBM-2 media and applied to confluent monolayers of GFP-ECs on the vessel lumen 4 days post-seeding. Separate scaffolds were incubated in the camptothecin solutions for 4 hr at 37°C followed by 2 rinses with PBS. Next, an Alexa Fluor® 647 annexin V conjugate (Invitrogen, Carlsbad, CA) solution was prepared in 1× annexin-binding buffer (Invitrogen, Carlsbad, CA) to a final concentration of 5% v/v. Annexin V specifically binds apoptotic cells only, with no binding associated with non-apoptotic cells. The scaffolds treated with camptothecin, and a non-camptothecin treated control scaffold, were then incubated in the annexin V solution for 15 minutes, after which the vessel lumens were imaged.

A 500 µm×250 µm area of the vascular lumen was scanned with the 473 nm laser to obtain a FOB image of GFP-EC distribution on the scaffold lumen. Next, to detect GFP-ECs that bound the far-red fluorescent annexin V conjugate, a 633 nm laser (05-LHR-121, CVI Melles Griot, Albuquerque, NM) was used to excite the apoptotic cells. A 647 nm longpass filter (Semrock, Rochester, NY) was used to block excitation light while allowing red fluorescence to reach the EM-CCD detector. The area previously imaged for GFP-ECs was then scanned with the 633 nm laser to obtain a FOB image of annexin V bound to apoptotic cells.

### Nondestructive quantification of graft endothelialization in response to scaffold fiber diameter

GFP-EC proliferation and coverage on full-vessel graft lumens was quantified in response to varying luminal electrospun fiber diameter. Full-vessel scaffolds with 3 different luminal electrospun fiber diameters (0.36 µm, 1.00 µm, and 3.32 µm) were fabricated by varying electrospinning parameters as outlined in Table 1. After cell seeding, three different 500 µm×250 µm areas of each scaffold were imaged daily and GFP-EC coverage was quantified for each scaffold group (n = 3 total per group) for 7 days. After imaging on day 7, the scaffolds were sectioned lengthwise and stained for F-actin and nuclei to compare to the FOB images at day 7. To accomplish this, the following steps were performed with PBS washes in between: samples were fixed in 3.7% paraformaldehyde (EMD chemicals, Gibbstown, NJ) for 20 min, permeabilized with 0.1% Triton X-100 (Sigma Aldrich) for 5 min, incubated in 1% bovine serum albumin (Santa Cruz Biotechnology, Inc., Santa Cruz, CA) for 30 min, incubated for 20 min in rhodamine phalloidin (Invitrogen) to stain for F-actin, and nuclei were counter stained with DAPI (Invitrogen). Cells were imaged using a conventional fluorescence microscope (Leica DMI6000-B, Wetzlar, Germany).

### Two-photon microscopy

The imaging depth of the FOB imaging system was directly compared to that of two-photon microscopy. Three electrospun PDLLA full-vessel scaffolds of varying thicknesses (95, 230, and 520 µm) were fabricated as described in section 2.1. The grafts were then sectioned lengthwise to allow direct access to image the lumen. GFP-ECs were then seeded onto the lumen of the vascular grafts at a density of 1.5×10^4^ cells/cm^2^. Cell seeded grafts were then allowed to incubate for 1 day before imaging. To image, sectioned grafts were placed into a culture dish with a 170 µm coverslip on which the scaffold rested. A two-photon microscope (Zeiss LSM 510, Carl Zeiss Inc., Germany) equipped with a 20× Plan-Apochromat objective (20x/0.8 N.A., Zeiss M27, Carl Zeiss Inc.) operating at 960 nm was then used to obtain images of GFP-ECs on the lumen surface by imaging through the electrospun vessel wall to resolve the GFP-ECs on the lumen surface. Additionally, images were obtained by placing the objective in direct-line-of-sight to the lumen surface to serve as control images.

### Statistical Analysis

All values are expressed as mean ± one standard deviation. Statistical analysis was performed using one-way analysis of variance (ANOVA) and significance was considered at p<0.05.

## Results and Discussion

### Nondestructive quantification of EC lumen coverage

**Figure 2:**
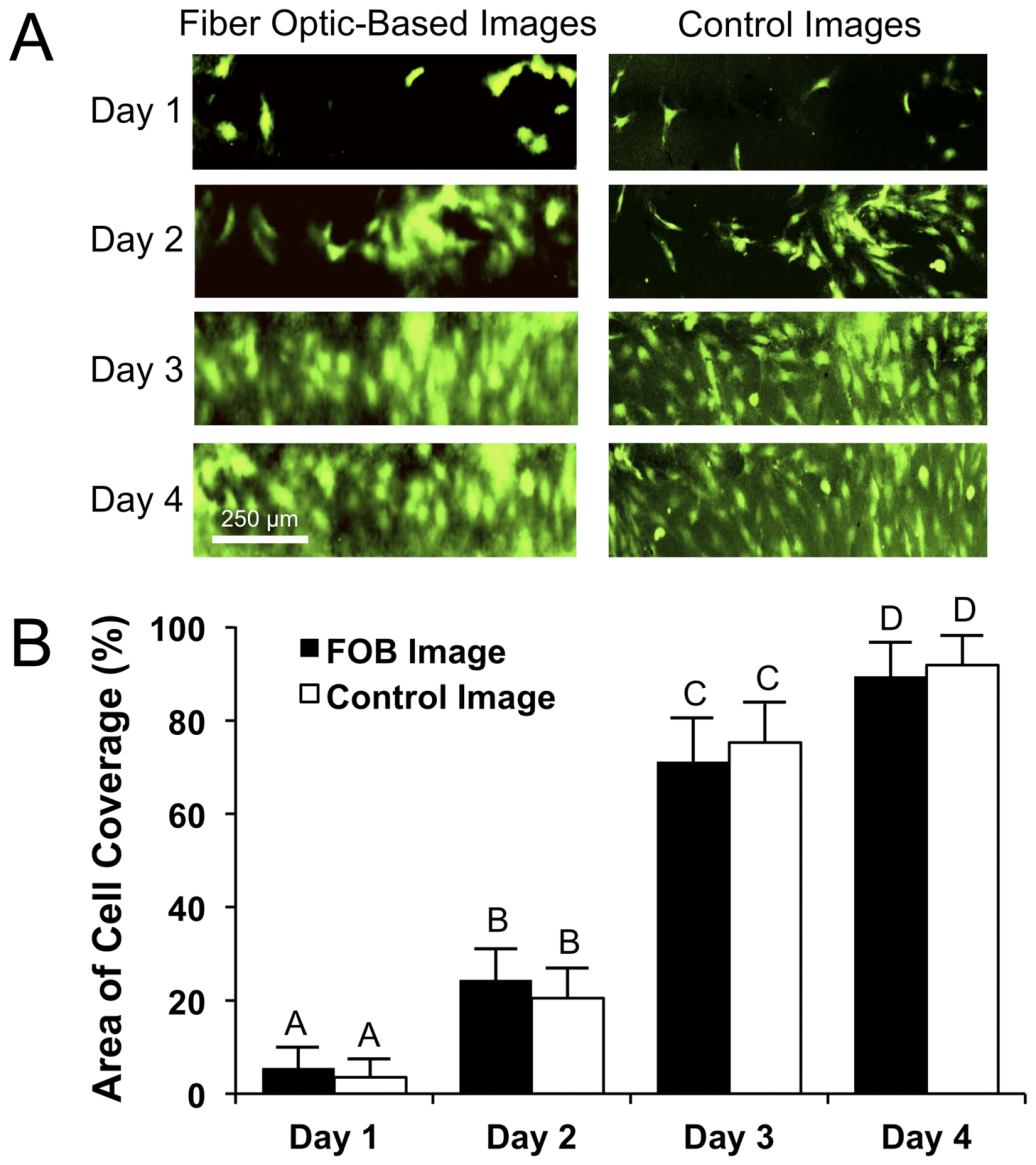
Quantification of GFP-EC proliferation and lumen coverage. A) A vascular scaffold sectioned lengthwise was seeded with GFP-ECs and 3 separate 1 mm×250 µm areas of the graft were imaged daily until the lumen reached confluency at Day 4 (representative images shown). B) Images were analyzed to quantify the amount of cell coverage on the vascular lumen (n = 3 images/time point). The results show that there is good agreement between amount of cell coverage for the FOB images, obtained through scaffold wall, and direct-line-of-sight control images at each time point. Data are presented as mean ± one standard deviation and values marked with the same letter are not significantly different (p<0.05).

Obtaining a confluent endothelium on the lumen of a vascular graft before implantation is vital to the success of the implant because thrombus formation is extremely sensitive to exposed surfaces [7]. Therefore, measurement of graft endothelialization is an important metric to determine the functionality and antithrombogenicity of the graft prior to implantation. The first set of experiments aimed to assess the feasibility of the FOB imaging method to noninvasively monitor endothelialization of the lumen surface of the vascular graft during incubation in a bioreactor. A half-vessel scaffold was seeded with GFP-ECs, placed in a bioreactor, and imaged over a 4-day period. Using the half-vessel configuration, we could simultaneously acquire a direct-line-of-sight control image of the lumen using conventional fluorescence microscopy (i.e. control camera). Figure 2A shows representative FOB and corresponding control images of the graft lumen for the duration of the study. The GFP-ECs have proliferated to cover the surface of the graft, and in 4 days have formed a nearly confluent endothelium with very few denuded regions. The FOB images closely resemble the control images in terms of overall cell distribution. To quantify the degree of lumen confluency, each image was analyzed for area of cell coverage (Figure 2B). The area of cell coverage for corresponding FOB and control images are not statistically different at each time point (p<0.05), demonstrating that the FOB imaging method provides an accurate assessment of endothelial coverage. Furthermore, the resolution of the FOB imaging method is sufficient to detect statistically different levels of endothelial coverage over time, even when the graft lumen becomes nearly confluent from day 3 to 4 (increase from ∼75% to ∼95% coverage).

### Nondestructive quantification of EC detachment

**Figure 3:**
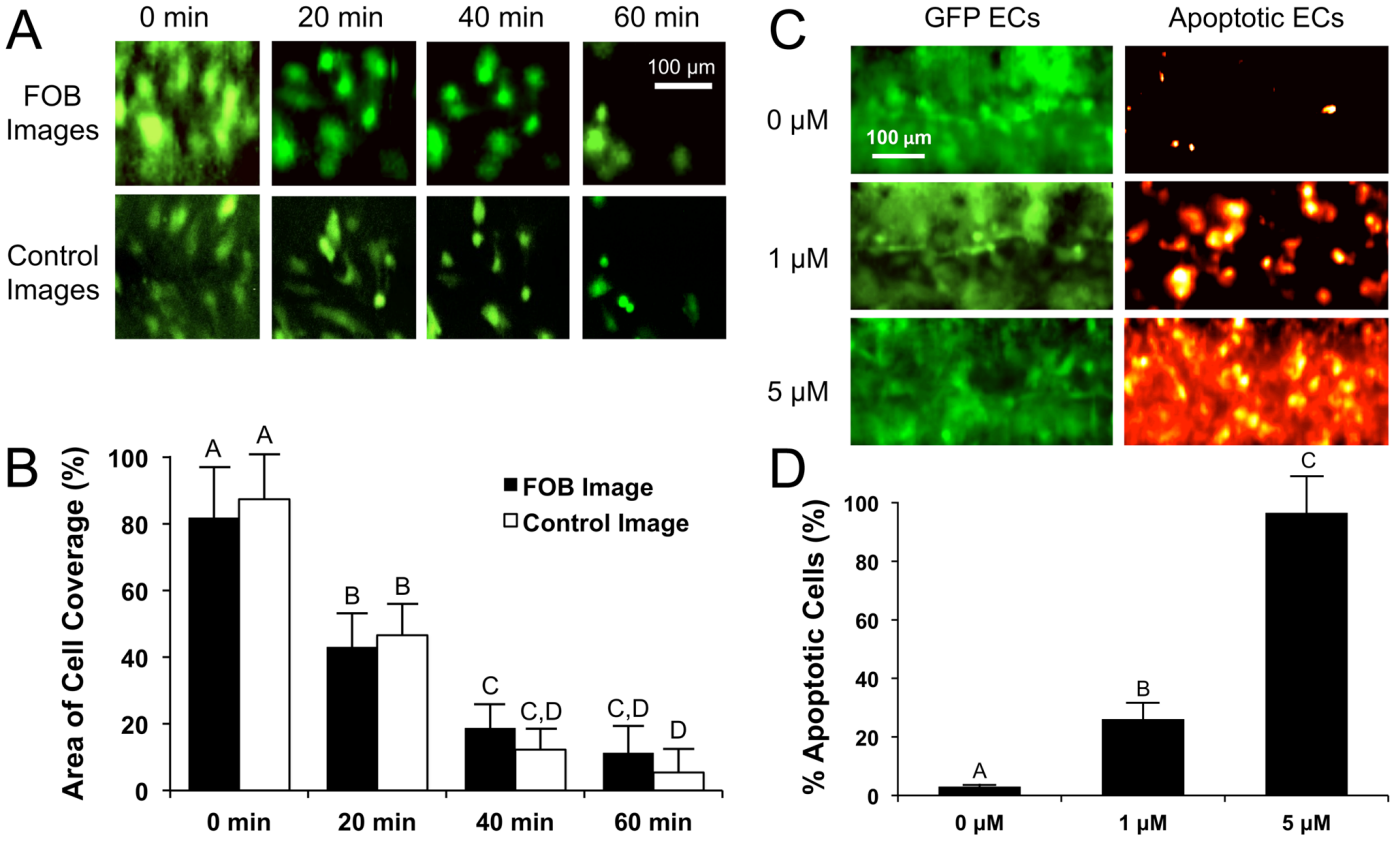
Quantification of GFP-EC detachment and apoptosis. A) Representative images of a near confluent endothelium that was subjected to a dilute concentration (1 µM) of trypsin EDTA to induce cell detachment from the lumen. An area of 250 µm×250 µm was continuously scanned to image GFP-EC detachment from the lumen over time. B) Extent of GFP-EC detachment from the lumen was quantified by analyzing the area of cell coverage from the fluorescent images (n = 3 images/time point). The FOB and corresponding control images had equivalent amounts of cell coverage at each time point. C) A near confluent endothelium was exposed to varying concentrations of camptothecin to induce GFP-EC apoptosis. A red fluorescent annexin-V conjugate was then added to visualize the extent of EC apoptosis on the vascular scaffold lumen. Three different 500 µm×250 µm areas were imaged to detect both GFP and red fluorescent annexin-V (apoptotic cells) on each scaffold for each condition. Results showed that varying levels of GFP-EC apoptosis could be noninvasively imaged and D) the fraction of apoptotic cells could be quantified through the scaffold wall using the FOB imaging method (n = 3 images/condition). Data are presented as mean ± one standard deviation and values marked with the same letter are not significantly different (p<0.05).

In this study, we tested if the FOB imaging method could successfully image and quantify EC detachment from the vessel lumen. Figure 3A shows a 250 µm×250 µm section of the vascular graft that was continuously imaged at 20 minute intervals after trypsin EDTA addition. At 0 min, the lumen surface is nearly confluent, and over time the ECs gradually detach from the surface. Similar to Figure 2A, there is close agreement between the FOB and control images, demonstrating that the FOB images accurately represent cell distribution on the lumen surface. These results were quantified by analyzing the images for area of cell coverage (Figure 3B) and show that the amounts of cell coverage between the corresponding FOB and control images are not statistically different at each time point (p<0.05). The high temporal resolution of the FOB imaging system allowed for dynamic assessment of EC detachment over time. Endothelial cell retention on the lumen of a bioengineered vascular graft is a key requirement to maintain long-term patency of the graft. EC detachment from the lumen surface of a vascular graft *in vivo* can potentially cause thrombus formation, leading to graft obstruction or embolism [6]. *In vitro*, EC detachment from the graft can be caused by a deficiency in adequate cell/biomaterial adherence, high shear stress, or as a result of shear induced apoptosis [23], [24]. The ability to noninvasively monitor EC detachment *in vitro*, especially for grafts undergoing fluid flow preconditioning, can provide real-time feedback on the integrity, confluency, and adhesiveness of the endothelium.

In addition to monitoring EC detachment, we are interested in determining the effect of fluid flow on vascular endothelialization and EC morphology. Traditional methods used to study the effect of fluid flow on ECs include the use of parallel plate flow chambers wherein cell culture media is perfused between two parallel substrates upon which the cells are cultured. These chambers deliver mechanical shear stress to the cultured cells by facilitating uniform laminar flow to develop across the cultured surface [25]. While useful for studying the effects of shear stress alone, these systems do not allow for combinatorial mechanical stimulation (i.e shear stress, hoop stress, and hydrodynamic pressure) that is known to occur in native arterial vessels. There is increasing data to support the hypothesis that not only shear stress modulates endothelial development and function, but also hoop stress caused by vessel stretch and hydrostatic pressure caused by pulsating blood flow [26], [27]. By incorporating the FOB imaging system in an intact vessel undergoing pulsatile flow *in vitro* to mimic vascular flow *in vivo*, we believe that the endothelium could be studied under a more physiological loading scenario where the lumen is subjected to all three modes of mechanical stimulation.

### Nondestructive quantification of EC apoptosis

Apoptosis, or programmed cell death, is a naturally occurring cell-death pathway that occurs in ECs and plays an important part in vascular tissue homeostasis. In this study, we induced various levels of EC apoptosis by exposing a near confluent endothelium to different concentrations of camptothecin, a well-known apoptosis-inducing agent [28]. To detect apoptotic cells, red fluorescent annexin-V conjugate was added, and the graft lumen was imaged for both GFP and far-red fluorescent annexin-V. Figure 3C (left column) shows that each graft had a near-confluent endothelium for each condition as evidenced by near complete GFP coverage. The control sample that was not exposed to camptothecin (0 µM) showed very little EC apoptosis, whereas the grafts exposed to 1 and 5 µM solutions experienced a dose dependent increase in cell apoptosis. Three sections of each vascular graft were imaged for both GFP and annexin-V and the resulting images were analyzed to quantify the fraction of apoptotic cells on the vessel lumen (Figure 3D). While EC apoptosis is a naturally occurring event in native vessels, premature or extensive apoptosis in engineered vascular grafts can be an indication of non-optimal culture or preconditioning methods [29], [30]. Recently, it has been shown that endothelial cell apoptosis, and subsequent cell death, can occur by biomaterial-induced toxicity [31], [32]. This phenomenon is caused by an inflammatory reaction due to the lack of, or inappropriate, contact of ECs with the biomaterial surface. Therefore, the ability to noninvasively detect apoptosis in engineered tissues during preconditioning is an important measure of tissue homeostasis and overall tissue health in response to preconditioning and cell/biomaterial interactions.

While we demonstrate here the capability to noninvasively assess EC apoptosis on the lumen of a vascular graft, the ability to image two distinct fluorophores could have multiple applications in monitoring the maturation of a bioengineered vascular graft. In this study, we show that it is possible to image two fluorophores using the FOB imaging system, namely co-localized GFP and far-red annexin-V conjugate. With this two-color imaging capability, it could be possible to detect two different color fluorescent proteins in the same cell simultaneously. For example, a cell could be co-transfected first with a constitutive fluorescent protein, such as GFP, for continuous visualization, and second with a red fluorescent protein (RFP) under control of a promoter for endothelial nitric oxide synthase (eNOS)–an enzyme that plays a critical role in nitric oxide production in response to shear stress. With this example, it would be possible to noninvasively monitor and quantify the effect of fluid flow preconditioning not only on endothelialization and endothelium integrity, but also on specific EC functions that are important for normal vascular function under fluid flow.

### Nondestructive quantification of graft endothelialization in response to scaffold fiber diameter

**Figure 4:**
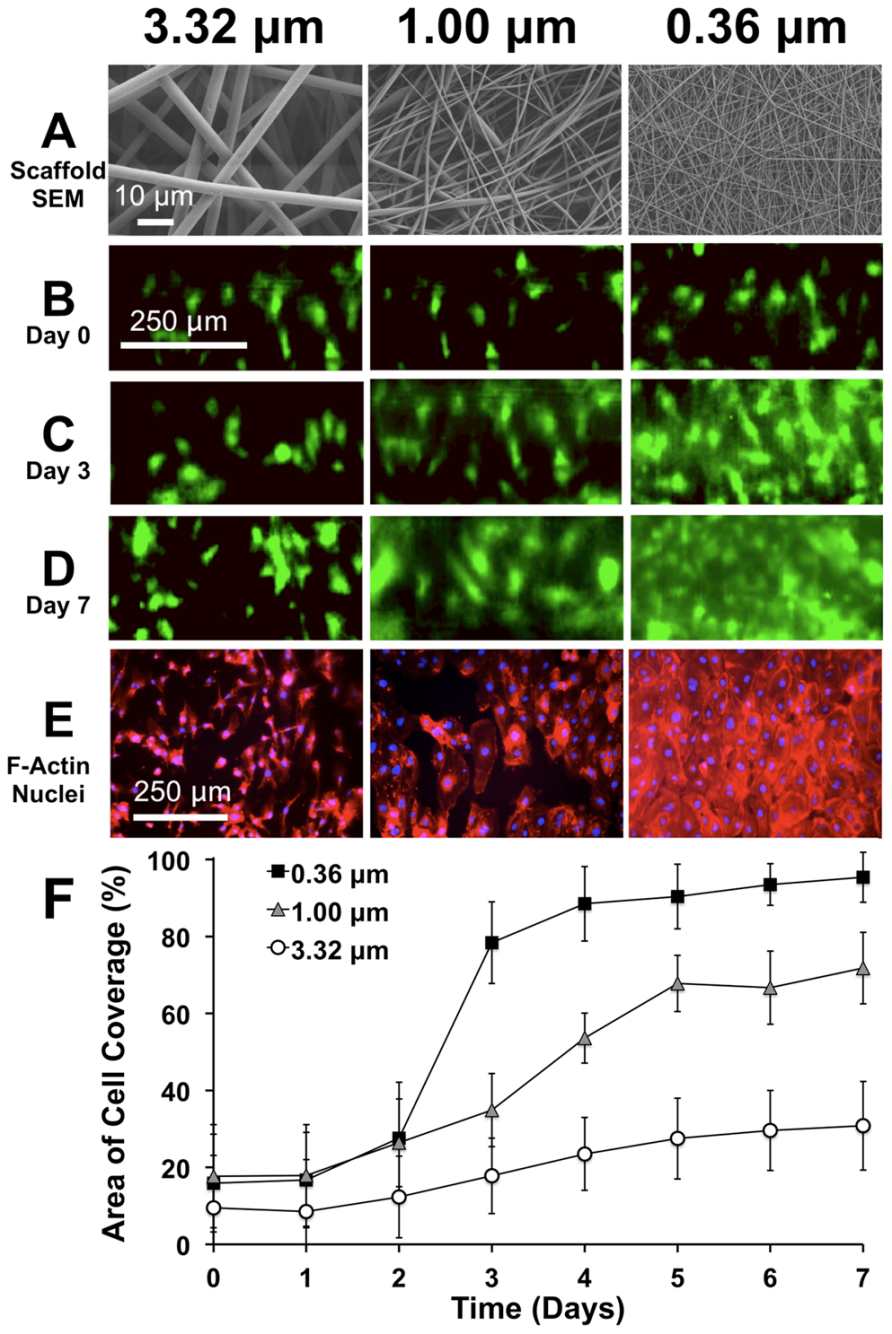
Effect of lumen topography on graft endothelialization. Full-vessel grafts with varied electrospun fiber diameters were fabricated to study the effect of lumen fiber diameter and varying topography on EC proliferation and endothelialization. A) Mean fiber diameters of 3.32 µm, 1.00 µm and 0.36 µm were electrospun by varying electrospinning parameters as shown in Table 1 (images captured using SEM). After cell seeding, three separate 500 µm×250 µm areas of each vascular graft were imaged daily to assess luminal coverage and EC growth over time. Representative FOB images of GFP-EC coverage are shown for each scaffold fiber diameter at B) Day 0, C) Day 3, and D) Day 7. E) After FOB imaging on Day 7, scaffolds were removed from the bioreactor, sectioned in half, stained for F-actin (red) and cell nuclei (blue), and imaged using a conventional florescence microscope to provide a comparison to the FOB images at Day 7. F) A total of n = 3 scaffolds for each fiber diameter were imaged, and n = 3 areas (500 µm×250 µm) for each scaffold, yielding a total of n = 9 images for each scaffold group every day. The FOB imaging method provided a noninvasive, accurate measurement of vascular graft lumen endothelialization over time. Data are presented as mean ± one standard deviation.

After validating the FOB imaging method using a half-vessel construct, we next tested the capacity of the FOB imaging system to measure the effect of varying surface topographies on lumen endothelialization of a full-vessel construct. Scaffold topography and architecture has been shown to affect EC proliferation, morphology, and formation of focal adhesions on the biomaterials surface [33], [34]. We therefore sought to determine if electrospun fiber diameter, and the resulting change in scaffold topography, would affect EC proliferation and the formation of an intact endothelium on the lumen surface. In these experiments, 3 different full-vessel scaffolds with varying lumen fiber diameter were fabricated to vary the lumen topography. Figure 4A shows SEM images of scaffolds for each fabrication condition (Table 1), which produced scaffolds with mean fiber diameters of 0.36±0.07 µm, 1.00±0.31 µm, and 3.32±0.63 µm. ECs were seeded onto the grafts and imaged at daily intervals for a 7-day period. For each fiber diameter, 3 scaffolds were fabricated and imaged at 3 different locations on each graft to produce n = 9 images for each condition per time point. Figures 4B, C, and D show representative images of each vascular graft condition at days 0, 3, and 7; respectively. EC lumen coverage appeared to be similar for each condition immediately after cell seeding on Day 0. After 3 days of incubation in the bioreactor, the graft lumen with the smallest fiber diameter (0.36 µm) appeared to have a greater extent of cell coverage than the other conditions, and at Day 7 these grafts had a near-confluent endothelium with very few void regions. Conversely, the grafts with the largest fiber diameter (3.32 µm) had minimal cell coverage even after 7 days of incubation. These images were analyzed to quantify the extent of cell proliferation and coverage on the surface of the vascular grafts in response to varying the surface topography. Each vascular graft lumen was noninvasively imaged daily with the FOB imaging system to provide a continuous trend of graft endothelialization over time (Figure 4F). The data shows that while coverage of the graft lumen increases over time for each condition, the graft with a 0.36 µm fiber diameter has the greatest proliferation rate and nearly encompasses (∼95%) the entire luminal surface by day 7. In comparison, the grafts with 1 µm and 3.32 µm fiber diameters show that the lumens achieve only approximately ∼70% and ∼30% coverage at day 7; respectively. These results are consistent with those of Ju *et al*. [35], who showed that human aortic endothelial cells could only form a confluent monolayer on PCL/collagen type 1 scaffolds with fiber diameters smaller than 1 µm in diameter. In the current study, grafts at day 7 were sectioned lengthwise and stained for F-actin and DAPI to compare to the FOB images at day 7. Figure 4D shows that the FOB images appear to be very similar to the stained images obtained with direct-line-of-sight fluorescence microscopy (Figure 4E). This data demonstrates that the FOB imaging system is sufficiently sensitive to accurately determine the rate of graft endothelialization and degree of EC confluency in response to varied scaffold properties during graft incubation in a bioreactor.

An important aspect is that the FOB imaging method has the capacity to assess EC distribution through a relatively thick wall of the vessel, and can therefore collect data on the lumen of the vessel without removal from the bioreactor or destruction of the sample. To collect the same information of lumen coverage as shown in Figure 2B in a tubular vascular graft using conventional fluorescence microscopy, 4 different scaffolds (one at each time point) would need to be seeded, incubated, and histologically sectioned to obtain identical information as we acquired with a single vessel using the FOB imaging system. Similarly, the data obtained in Figure 4F would require a total of 72 scaffolds (n = 9 different scaffolds at each time point) using conventional microscopy as compared to repeatedly imaging the same 9 scaffolds at each time point using the FOB imaging system. Conventional fluorescence microscopic techniques would require different samples at each time point since the vessel would have to be histologically sectioned to image the lumen, thus destroying the scaffold. Direct-line-of-sight imaging such as this is necessary since the wall of the vascular graft is far too thick and optically scattering to obtain an image of the endothelium through the vessel wall using microscopic techniques such as confocal laser scanning microscopy [21].

### Two photon microscopy and comparison to conventional imaging methods

**Figure 5:**
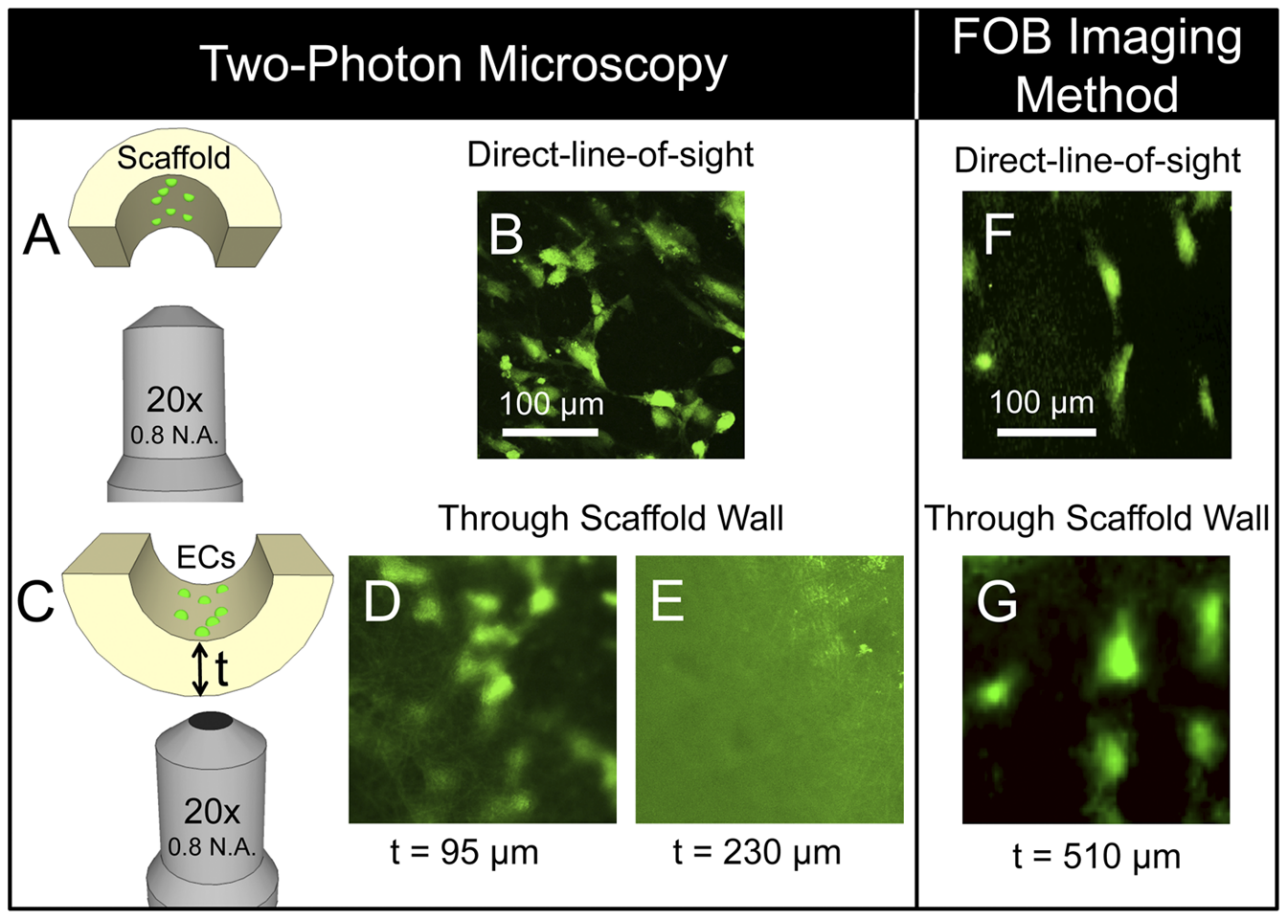
Imaging penetration depth: comparison of FOB imaging method to Two-photon microscopy. A) EC-seeded half-vessel scaffolds were imaged with a two-photon microscope in direct-line-of-sight to the lumen. B) Representative image of GFP-ECs in direct-line-of-sight to the vessel lumen. C) The two-photon microscope was then used to image GFP-ECs on the lumen through the wall of vascular scaffolds of varying thicknesses (t = 95, 230 and 520 µm). Images of GFP-ECs on the vessel lumen through a D) 95 µm and E) 230 µm thick scaffold. Although cells were visible through the 95 µm thick scaffold, GFP-ECs could not be resolved through the 230 and 520 µm thick scaffolds. The FOB imaging method, however, was capable of resolving individual GFP-ECs through a ∼510 µm thick scaffold wall (G) and the overall cell distribution of the same region of interest matched very closely to the direct-line-of-sight fluorescence image (F).

Two-photon microscopy, or multi-photon microscopy, is a powerful imaging technique used to image deep within biological tissues or scaffolds with high resolution [36]. Currently, there is no other diffraction-limited optical imaging system that can reach the imaging penetration depth of multi-photon microscopy at a given resolution [37]. We therefore compared the imaging penetration depth of the FOB imaging system to that of two-photon microscopy. To accomplish this, 3 different PDLLA vascular scaffolds with varying wall thickness (95, 230, and 520 µm) were fabricated using the electrospinning approach as described earlier. The scaffolds were sectioned lengthwise, seeded on one side of the scaffold with GFP-ECs, and then imaged using a two-photon microscope. The scaffolds were placed in a cell culture dish with glass coverslip bottom to allow imaging access within the working distance of the objective. First, the scaffold lumens were imaged in direct-line-of-sight to the lumen to ensure that ECs were on the lumen surface (Figure 5A,B). Next, the scaffolds were positioned such that the exterior surface of the scaffold wall faced the optical objective to mimic a situation in which a two-photon microscope would be used to image through the vascular scaffold wall to view GFP-ECs on the lumen (Figure 5C). Figure 5D shows an image obtained through a 95 µm thick PDLLA electrospun scaffold to view the GFP-ECs on the lumen surface. In this image, the resolution of the cells is diminished as compared to the direct-line-of-sight view in Figure 5B. This reduction in imaging resolution is likely due to the highly scattering nature of the electrospun material. Figure 5E shows that the cells could not be resolved when imaging through the 230 µm or 520 µm electrospun PDLLA scaffolds (520 µm scaffold image not shown) using two-photon microscopy. The FOB imaging system, however, was capable of imaging through the ∼500 µm thick PDLLA scaffold with sufficient resolution to accurately identify the distribution of GFP-ECs (Figures 5F,G).

This experiment illustrates the difficulty of performing nondestructive imaging of a bioengineered vascular graft endothelium while housed in a bioreactor using conventional microscopy techniques. Confocal or two-photon microscopy are options to image the endothelium, however, due to the photon-scattering nature of the highly porous scaffold, it is exceedingly difficult to image through the thick vessel wall. In the present study, we demonstrated that two-photon microscopy only had an effective imaging depth of approximately 95 µm through the highly opaque electrospun PDLLA scaffold. Electrospun vascular grafts used for bypass grafting typically range from 300 µm to 1 mm in thickness to sustain the physiological forces associated with blood pressure [5], [35], [38]. It is therefore impossible to use conventional microscope imaging techniques to penetrate through the wall of these vessels to noninvasively image the endothelium.

A potential alternative to using conventional microscope-based systems to image the endothelium are endomicroscope-based probes. In this approach, the endomicroscope could be inserted into the lumen of the vessel through the bioreactor flow chamber. Several groups have recently used endomicroscopes on the order of 1 mm in diameter to image cells and tissues both *in vitro* and *in vivo*. One notable example is a rotational side-view endomicroscope developed by Kim *et al* that is capable of imaging the gastrointestinal and respiratory tracts of mice *in vivo* with single-cell level resolution and a 250 µm×250 µm field of view [39]. This imaging technique could potentially be used to assess the endothelium of a bioengineered vascular graft *in vitro*, however issues with sterility may limit its use due to repeated insertion into the bioreactor at evaluation intervals. Moreover, insertion of the endomicroscope into the lumen will likely disrupt the flow profile during fluid flow preconditioning. Therefore, this imaging method may not be suitable for dynamic assessment of a preconditioning vessel housed within a fluid flow bioreactor.

The FOB imaging system, alternatively, uses a micro-imaging channel embedded in the wall of the scaffold through which the excitation fiber optic is inserted and GFP-EC fluorescence is captured from outside the bioreactor using an EM-CCD detector. This allows for nondestructive, dynamic imaging of the endothelium of the vascular graft with no impact on the vascular scaffold or biological system in the bioreactor. Additionally, the FOB imaging method is capable of continuous scanning and subsequent image acquisition. This provides real-time data that could not otherwise be obtained using conventional, static fluorescence imaging using conventional microscopes. In Figures 2 and 4, the vascular grafts were imaged at daily intervals to assess graft endothelialization; however, it is possible to provide continuous scanning and image acquisition with the FOB imaging system. To demonstrate this capability, a 500 µm×250 µm section of a full-vessel scaffold was sequentially imaged at 2 hr intervals for a 3-day period. Supplemental video S1 shows the GFP-ECs on the scaffold lumen and corresponding plot of GFP-EC coverage over time. In this video, GFP-ECs are actively migrating along the surface of the lumen and each image was analyzed to provide a real-time assessment of EC coverage, which in this case increases with incubation time. It should be noted that the FOB imaging system is fully automated and does not require any manual adjustments during semi-continuous imaging. This capability of the FOB imaging system could be used as a “hands-off” approach to study EC proliferation rates and endothelialization with greater temporal resolution than could be obtained using conventional fluorescence microscopy. Additionally, the FOB imaging system could potentially be used to monitor EC migration along the vascular graft lumen in response to various stimuli, such as varied fluid flow regimes–an impossible task using conventional fluorescence microscopy and a difficult task for endomicroscopy techniques.

## Conclusion

In this study, we demonstrated the capability of the FOB imaging system to provide nondestructive, real-time data regarding endothelialization of a bioengineered vascular graft *in vitro*. The dynamic capabilities of this method allowed visualization and quantification of endothelial cell proliferation, coverage, detachment, and apoptosis. Additionally, we showed that the FOB imaging method is sufficiently sensitive to detect varied endothelialization rates in response to varied biomaterial topographies. Finally, we demonstrated that the FOB imaging system had a much greater imaging depth than that of two-photon microscopy. This method has clear advantages over endpoint testing to assess bioengineered vascular graft endothelium maturation *in vitro* and has the potential to be used to assess other endothelialized bioengineered tissues as well.

## Supporting Information

Video S1 **Dynamic imaging of graft endothelialization.** A full-vessel scaffold was seeded with GFP-ECs and a 500 µm×250 µm section of the lumen was sequentially imaged at 2 hr intervals for a 3-day period. The video shows the scaffold lumen and corresponding plot of GFP-EC coverage over time. In this video, GFP-ECs are actively migrating along the surface of the lumen and each image can be analyzed to provide a real-time assessment of EC coverage.
(MOV)
